# The Use of AI in Mental Health Services to Support Decision-Making: Scoping Review

**DOI:** 10.2196/63548

**Published:** 2025-01-24

**Authors:** Hassan Auf, Petra Svedberg, Jens Nygren, Monika Nair, Lina E Lundgren

**Affiliations:** 1 Halmstad University School of Health and Welfare Halmstad Sweden; 2 School of Business, Innovation and Sustainability Halmstad University Halmstad Sweden

**Keywords:** artificial intelligence, AI, mental health, decision-making, shared decision-making, implementation, human-computer interaction

## Abstract

**Background:**

Recent advancements in artificial intelligence (AI) have changed the care processes in mental health, particularly in decision-making support for health care professionals and individuals with mental health problems. AI systems provide support in several domains of mental health, including early detection, diagnostics, treatment, and self-care. The use of AI systems in care flows faces several challenges in relation to decision-making support, stemming from technology, end-user, and organizational perspectives with the AI disruption of care processes.

**Objective:**

This study aims to explore the use of AI systems in mental health to support decision-making, focusing on 3 key areas: the characteristics of research on AI systems in mental health; the current applications, decisions, end users, and user flow of AI systems to support decision-making; and the evaluation of AI systems for the implementation of decision-making support, including elements influencing the long-term use.

**Methods:**

A scoping review of empirical evidence was conducted across 5 databases: PubMed, Scopus, PsycINFO, Web of Science, and CINAHL. The searches were restricted to peer-reviewed articles published in English after 2011. The initial screening at the title and abstract level was conducted by 2 reviewers, followed by full-text screening based on the inclusion criteria. Data were then charted and prepared for data analysis.

**Results:**

Of a total of 1217 articles, 12 (0.99%) met the inclusion criteria. These studies predominantly originated from high-income countries. The AI systems were used in health care, self-care, and hybrid care contexts, addressing a variety of mental health problems. Three types of AI systems were identified in terms of decision-making support: diagnostic and predictive AI, treatment selection AI, and self-help AI. The dynamics of the type of end-user interaction and system design were diverse in complexity for the integration and use of the AI systems to support decision-making in care processes. The evaluation of the use of AI systems highlighted several challenges impacting the implementation and functionality of the AI systems in care processes, including factors affecting accuracy, increase of demand, trustworthiness, patient-physician communication, and engagement with the AI systems.

**Conclusions:**

The design, development, and implementation of AI systems to support decision-making present substantial challenges for the sustainable use of this technology in care processes. The empirical evidence shows that the evaluation of the use of AI systems in mental health is still in its early stages, with need for more empirically focused research on real-world use. The key aspects requiring further investigation include the evaluation of the use of AI-supported decision-making from human-AI interaction and human-computer interaction perspectives, longitudinal implementation studies of AI systems in mental health to assess the use, and the integration of shared decision-making in AI systems.

## Introduction

### Background

There has been an increasing interest in the use of artificial intelligence (AI) systems in health care in recent years as part of supporting health care professionals and individuals with health problems to facilitate care processes. This technology is taking advantage of the abundance of health care data to assess and support decision-making across multiple dimensions. Numerous studies have investigated the prospective utility of AI systems in augmenting decision-making across various health care domains. These encompass early detection, diagnostics, treatment modalities, and self-care initiatives [1-3]. While extensive research has examined the ethics, performance, and potential applications of AI models [4-6], there remains a notable gap in the literature regarding the integrative and empirical use of implemented AI systems in health processes.

AI systems for decision-making are equipped to enhance health care and self-care processes. These systems not only have the potential to improve the efficiency of health care processes but also act as a bridge, integrating health care with self-care by enabling seamless information exchange between the 2 domains [7]. From a health care viewpoint, AI systems hold transformative potential for the future, particularly in the identification and diagnosis of health problems, as it can introduce more objectivity and precision [8]. Self-care, in contrast, refers to the personal management of health and daily life activities outside the formal health care system, while self-help encompasses the use of external resources for the management of health and well-being, both within and beyond the formal health care system [9].

From the standpoint of clinical workflows and end users, AI systems present several advantages, including personalized decision-making, enhanced patient-physician communication, and support for shared decision-making (SDM) [10-12]. However, concerns have been raised by both clinicians and patients regarding the widespread adoption of AI systems, underscoring the need for cultural adjustments within health care to facilitate acceptance [10]. Clinicians have expressed reservations about integrating AI systems into decision-making processes, given the challenges around accepting a second opinion without validation from another clinician [12]. Beyond issues of acceptability, the implementation of AI in SDM poses various challenges, including potential delays in decision-making, communication barriers between health care professionals and patients, and uncertainty surrounding the role of AI in decision-making [13]. Research into the use of AI systems in the context of SDM is still in its nascent stages, emphasizing the need for further exploration into how AI systems can effectively support SDM processes in the future [14].

The potential use of AI for SDM holds particular significance in the context of mental health care given the challenges in the identification and management of mental health problems faced by the contemporary mental health care institutions. However, the complex and heterogeneous nature of mental health problems presents considerable obstacles for AI systems in delivering accurate diagnostics, effective treatment guidance, and tailored interventions. Instead, AI systems currently demonstrate greater efficacy in aiding the identification of symptoms of mental health problems rather than diagnosing specific disorders [10,15]. Given this context, it becomes imperative to understand the interplay between the complexities of mental health problems and AI decision-making support mechanisms, emphasizing the incorporation of the perspectives of end users. Such an inclusive approach is essential for guiding the effective implementation of AI systems in mental health settings [16]. Numerous studies underscore the necessity for a nuanced understanding of AI system use within real-world mental health care to inform future implementation strategies [1,17-19].

### Objectives

Despite the potential promises and inherent challenges associated with AI systems in supporting decision-making processes within mental health care, there remains a notable research gap concerning their practical utility. Fundamental questions persist regarding the types of decisions AI systems can support in real-world environments, the extent to which these systems engage patients in the decision-making process, and the methodologies used for their evaluation. Given this backdrop, this study aims to explore the empirical evidence for the use of AI systems to support different types of decision-making in mental health. This includes how it has been researched, applied, and evaluated for use and implementation, contributing to the understanding and improvement of future design, development, and implementation of AI systems in mental health.

## Methods

### Study Design

A scoping review approach was considered the preferable method to address the explorative nature of the study and to systematically map the available empirical studies within the study domain. The design of this scoping review was guided by the 5-phase framework put forward by Arksey and O’Malley [20] to ensure quality and a systematic approach to the study. The framework phases included defining the research questions (RQs); identifying relevant studies; selecting the study; charting the data; and collating, summarizing, and reporting results. The reporting process was conducted following the PRISMA-ScR (Preferred Reporting Items for Systematic Reviews and Meta-Analyses Extension for Scoping Reviews) checklist [21], as shown in Multimedia Appendix 1.

### Identifying the RQs

A multidisciplinary research team comprising experts from health care, nursing, AI systems, and implementation research engaged in discussions in the initial phase of the study to identify the scope of the research and RQs. Four main concepts were defined to guide the study: mental health, AI, decision-making, and implementation (Table 1).

**Table 1:** Operational definitions of the main concepts used in the article.

Concept	Operational definition	Specification
Mental health	It is conceptualized as including mental health problems, mental illnesses, and disorders for patients and individuals seeking care [22].	This enabled the exploration of broader types of users with mental health problems, not only patients in clinical settings but also individuals seeking care in a nonclinical environment. The term patient is used to refer to a person receiving or registered with a health care provider. The term individuals refers to people seeking care regardless of being registered in health care.
AI^a^ systems	The AI concept refers to systems described to use AI types relevant to health care, including ML^b^, neural networks, deep learning, computational intelligence, supervised ML, or robotics in connection to supporting decision-making utility [23].	Exploring the AI models as part of a system facilitates understanding the processes within which the AI is integrated into the care settings.
Decision-making	Decision-making is defined as a cyclical process from situation awareness to acting on a decision [24,25]. Decision-making support provided by the AI systems are systems that “provide clinicians, staff, and patients with knowledge, patient-specific information, and recommendations” and are “designed to assist decision-makers and interactively support all phases of a human decision-making process.” [26,27]. Decision-making in mental health may include a collaboration between the patients and health care professionals to reach a decision. Shared decision-making in this study is defined as “an approach where clinicians and patients make decisions together using the best available evidence” [28].	This concept allowed the investigation of AI systems from a decision-making support utility perspective in health care and self-care settings.
Implementation	The criteria for the level of implementation of AI systems in practice were based on a TRL^c^ of ≥6 [28]. A TRL of 6 indicates that the technology has advanced beyond the basic development phase and is now being demonstrated in a relevant real-world or simulated environment, meaning it is now ready for operational testing within its intended context.	This study focuses on researching AI systems specifically in terms of their use and implementation within care settings, with an emphasis on how these technologies are used and adopted in practice. Thus, AI systems with a TRL of ≤5 were excluded, as they are not sufficiently developed to allow end-user interaction or for meaningful evaluation in real-world care environments.

^a^AI: artificial intelligence.

^b^ML: machine learning.

^c^TRL: technology readiness level.

The following three RQs were formulated to address the aim of the study:

1. What are the characteristics of research on AI systems used in relation to support decision-making in mental health?
2. Which types of technologies, decisions, actors, and user flows of AI systems to support decision-making are described?
3. How were the AI-based decision support systems evaluated in research in the mental health context, and what discernible consideration might enable or hinder the adoption or implementation of these systems?

### Identifying Relevant Studies

A search strategy was developed within the research team with the assistance of a librarian. Literature searches were carried out using the 5 main health research databases: PubMed, Scopus, PsycINFO, Web of Science, and CINAHL. Keywords used in the searches were guided by the following concepts and Medical Subject Headings terms: *mental health*, *artificial intelligence*, *decision-making*, and *implementation*. Synonyms were joined by the Boolean operator OR; next, we combined the search strings for each keyword with the Boolean operator AND (Multimedia Appendix 2). Eligibility criteria were followed for the search and study selection, as shown in Textbox 1.

**Eligibility criteria.**

**Inclusion criteria**

- Written in English
- Peer-reviewed
- Publication date between January 2011 and September 2022
- All artificial intelligence (AI)–based decision support systems intended for use in relation to mental health interventions
- AI tools with user interaction with patients or health professionals aiming to support decision-making
- All mental health care settings and providers
- Empirical study design

**Exclusion criteria**

- Interventions that use AI for other purposes than to support decision-making
- Interventions that deliver support for other than mental health professionals and care receivers
- Proof-of-concept, viewpoints, or validation studies not related to the implementation and use of AI

### Study Selection (Screening)

All articles identified from the searches were uploaded to EndNote (version 20.1; Clarivate) to remove duplicates. The remaining articles were then uploaded to Rayyan (Rayyan Systems Inc), a web-based tool used to screen articles on the abstract level and identify eligible studies for full-text screening. Rayyan was selected as it provides a systematic approach and facilitates organized teamwork [29]. In the first phase of screening, researchers (HA and MN) reviewed the abstracts and held regular follow-up meetings to ensure criteria consistency. In the second phase, full-text articles were reviewed by 2 reviewers in the research team and screened to meet the eligibility criteria and address the RQs. Throughout both phases, articles where there were uncertainties about meeting the inclusion criteria were discussed among the full research team until a consensus was reached. The screening process followed a decision tree process for excluding the studies that did not meet the inclusion criteria, then illustrated in a PRISMA (Preferred Reporting Items for Systematic Reviews and Meta-Analyses) diagram.

### Charting the Data (Data Extraction)

A template sheet was developed to extract data from each of the included articles regarding the characteristics of research to respond to the first RQs, including the author’s name, publication date, title, aim, country, study design, care settings, mental health problems, and types of AI. A descriptive summary was performed to answer RQ1 concerning the characteristics.

### Collating, Summarizing, and Reporting Results

The reporting of the results for RQ2 was performed in 3 parts. First, we categorized the AI systems described in the included articles according to their utility in supporting decision-making in mental health. Second, we mapped the AI system’s utility of supporting decision-making in relation to the end user’s interaction and use. This mapping was carried out in terms of types of interaction and decision-making stages as per a conceptual model [24,25], which describe decision-making as a cyclical process spanning from situational awareness to the execution of decisions. Third, we conducted an explorative analysis, mapping data flow and user interaction in relation to the AI system’s basic structure as elucidated by Sremac et al [30], comprising 3 primary phases: training data, AI model, and AI output (decision). In the identified articles, the study by Sadeh-Sharvit et al [31] did not provide sufficient data in the reporting for RQs 2 and 3.

An abductive analysis was conducted for RQ3 [32] combining inductive and deductive reasoning when looking at the data by firstly conducting inductive thematic analysis and connecting the emerged themes deductively to the human, organization, and technology-fit (HOT-fit) framework [33]. This framework provides 3 essential components when an AI system is being used; the human factor focuses on the individual user experience of the AI system; the organization factor focuses on the structure and environment evaluation of use; and the technology factor focuses on the quality of use of the system and the information provided by the AI system. These factors consist of subcategories or dimensions, and each dimension includes several elements. First, the content in each article was collated by the first author (HA) and inductively abstracted into themes. The framework was then used to deductively map the themes into the factors and their included elements. Two new elements, *trustworthiness* and *explainability*, were found in the abductive analysis and were included because they were relevant to the study objectives. Explainability refers to the ability of the AI system to provide clear and understandable reasoning of its output or decision support provided to the intended end users [34]. Trustworthiness refers to the extent to which the AI system is trusted by the end users to use, including being reliable, ethical, and dependable for use and in supporting decision-making [35]. The analysis was iteratively and repeatedly discussed between all the authors until consensus was achieved.

## Results

### Overview and Study Characteristics

**Figure 1:**
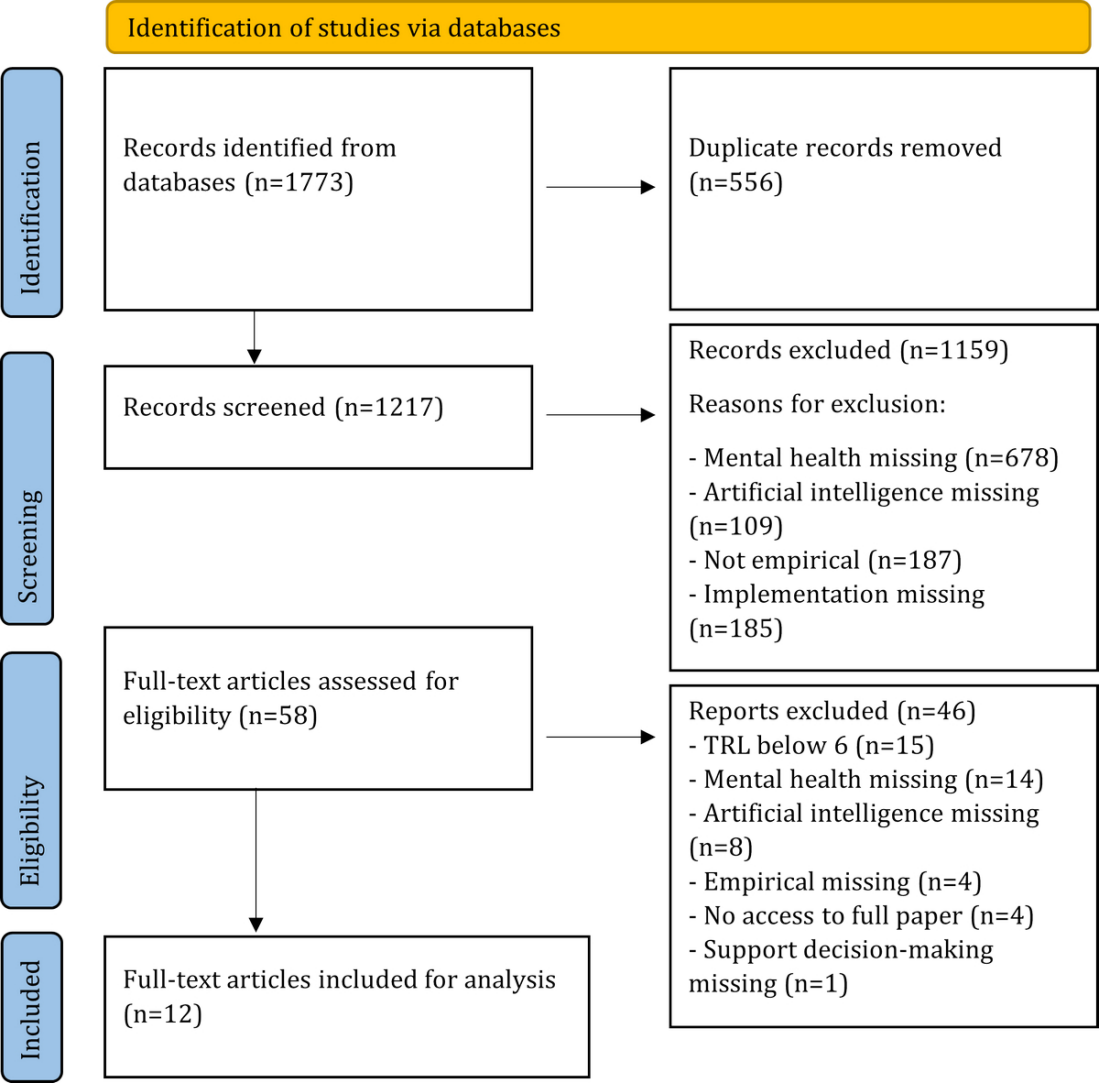
PRISMA (Preferred Reporting Items for Systematic Reviews and Meta-Analyses) flowchart—study search and selection process. TRL: technology readiness level.

The literature search resulted in 1773 articles between the years 2011 and 2022. Following the removal of duplicates, 1217 articles remained for abstract-level screening. Of these, 1159 (95.23%) articles were excluded based on the inclusion and exclusion criteria, leaving 58 (4.77%) articles for full-text screening. A total of 12 (0.99%) articles were found to meet our study eligibility criteria after the completion of the screening process as seen in the PRISMA flowchart (Figure 1).

The articles included were published between 2020 and 2022, and a variety of designs were used (Table 2). Most of the articles were from the United States (7/12, 58%); 25% (3/12) were from Canada, 8% (1/12) were from the United Kingdom, and 8% (1/12) were from China. The included studies encompass 3 settings of investigation: health care, self-care (remotely), and simulation environment. The health care studies took place in primary care, emergency department, and specialized mental health care. The self-care studies focused on AI systems using mobile apps or wearables with sensors. Simulation studies were conducted in the psychiatry department within a university-based setting. The spectrum of severity and types of mental health problems found to be supported by the AI systems varied. Depression was the most common mental health problem targeted by the AI system support (5/12, 42%), followed by substance misuse (2/12, 17%), autism spectrum disorder (2/12, 17%), suicide and mental health crisis (2/12, 17%), and psychotherapy for different mental health problems (1/12, 8%).

**Table 2:** General characteristics of the included articles (N=12).

Study, year, and country	Study aim	Study design	Care setting	Mental health problems	Type of AI^a^ (ML^b^, DL^c^, etc)
Dosovitsky et al [36], 2020; United States	To understand how users engage and are redirected through a chatbot for depression (Tess) to provide design recommendations.	Descriptive study	Self-care and online access	Depression	Not specified (using AI or AI model)
Benrimoh et al [37], 2021; Canada	To explore the use of a simulation center environment in evaluating the usability of (Aifred), particularly its impact on the physician–patient interaction.	Simulation-based study design	Simulation in a specialized care context (research and development)	Depression (major depressive disorder)	DL
Dimeff et al [38], 2021; United States	To evaluate the feasibility, acceptability, and effectiveness of (Jaspr Health) among suicide adults in emergency departments.	Pilot RCT^d^	Emergency department	Suicide	Not specified (using AI or AI model)
Jacobs et al^e^ [39], 2021; United States	To investigate the influence of ML and AI on clinician decisions in major depressive disorder treatment.	Experimental study design	Primary and specialized health care	Depression (major depressive disorder)	ML
Popescu et al^e^ [40], 2021; Canada	To examine the feasibility of an AI-powered CDSS^f^, which combines the operationalized 2016 Canadian Network for Mood and Anxiety Treatments guidelines with a neural network–based individualized treatment remission prediction.	Longitudinal feasibility study	Primary and specialized health care	Depression (major depressive disorder)	DL
Prochaska et al [41], 2021; United States	To evaluate the efficacy and use of Woebot-SUDs^g^ in managing SUD relative to a waitlist group in an RCT.	RCT	Self-care and online access	SUD	Not specified (using AI or AI model)
Prochaska et al [42], 2021; United States	To examine the feasibility, acceptability, and preliminary efficacy of Woebot-SUDs in managing SUD.	Development and usability study	Self-care and online access	SUD	Not specified (using AI or AI model)
Deng et al [43], 2022; China	To evaluate the effectiveness of sensory management recommendation system device for children with autism.	Experimental study	Self-care (wearables and app)	Autism	ML
Garriga et al [44], 2022; United Kingdom	To explore the added value of an implemented ML model in a clinical context.	Prospective cohort study	Specialized and primary care	A range of mental health problems	ML
Megerian et al [45], 2022; United States	To test the accuracy of an AI-based software as a medical device designed to aid primary care health care providers in diagnosing autism spectrum disorder.	A double-blinded, multisite, prospective, active comparator cohort study	Primary care	Autism	ML
Sadeh-Sharvit et al [31], 2022; United States	To explore therapists’ use of a standard component that is pertinent across most behavioral treatments—prompting clients to summarize their treatment session as a means for consolidating and augmenting their understanding of the session and the treatment plan.	Retrospective study	Specialized care—behavioral health treatments	A range of mental health problems	ML and NLP
Tanguay-Sela et al [46], 2022; Canada	To evaluate the utility of a CDSS as perceived by physicians participating in simulated clinical interactions.	Simulation-based study design	Simulation in a primary and specialized care context (research and development)	Depression (major depressive disorder)	DL

^a^AI: artificial intelligence.

^b^ML: machine learning.

^c^DL: deep learning.

^d^RCT: randomized controlled trial.

^e^Jacobs et al [39] and Popescu et al [40] did not explicitly state the study aim in the articles.

^f^CDSS: clinical decision support system.

^g^SUD: substance use disorder.

### Decision Support Utility, Data Flow, and User’s Interaction With the AI System

#### Decision Support Utility

In our findings, 3 types of AI systems emerged based on their decision support utility and the form of output they provide to support decision-making in mental health, as shown in Multimedia Appendix 3. These three types are as follows: (1) *diagnostic and predictive AI*, including AI systems providing support in the identification of mental health states with diagnostic or prognostic output in binary or categorical forms; (2) *treatment selection AI*, including AI systems providing options for treatment with information explaining each option, designed to assess health care practitioners and patients in the therapy selection process; and (3) *self-help AI*, including AI systems designed to support patient and individuals in managing their own mental health, either in health care setting or through self-care practices. The self-help AI in the reviewed studies were all based on conversational agents, which address various mental health problems through psychoeducation and behavioral therapy methods.

**Figure 2:**
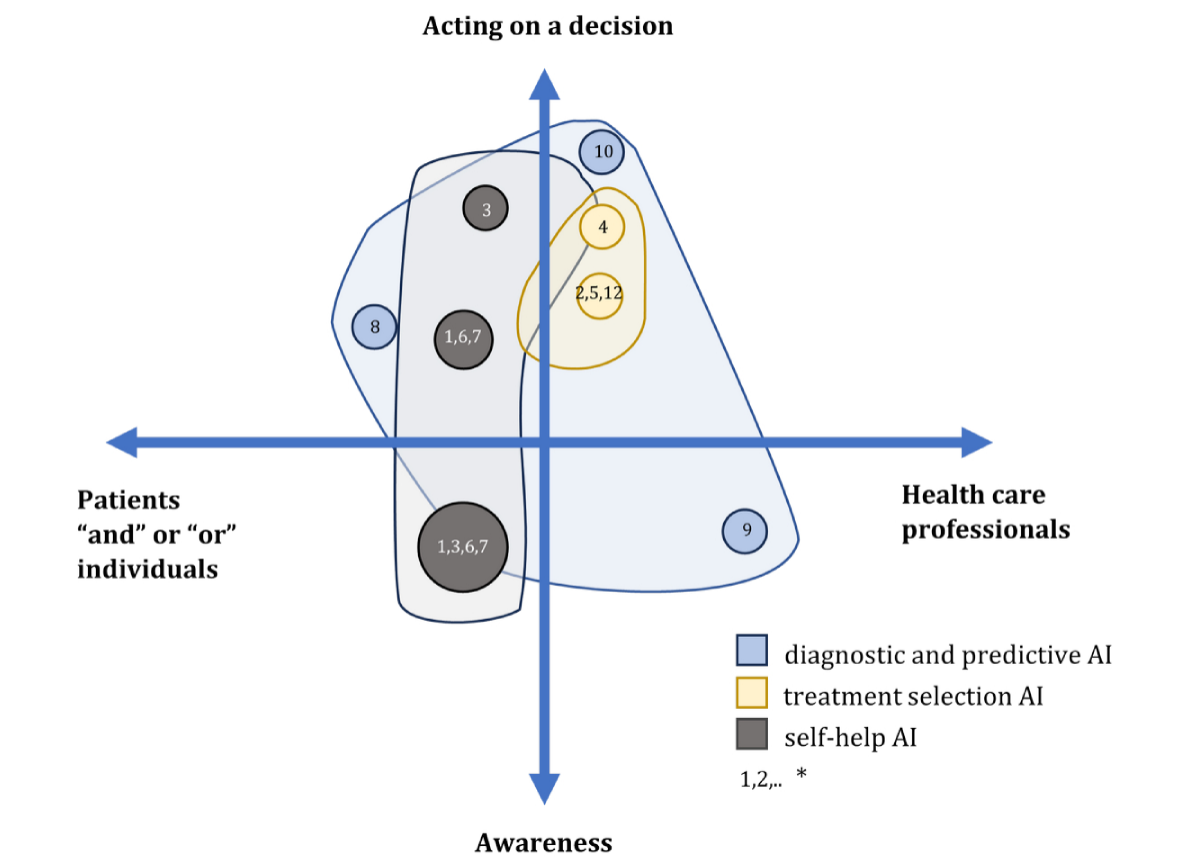
Mapping the decision-support utility of the artificial intelligence (AI) systems on two dimensions: (1) the extent of actionable output the AI system provides and (2) the types and variety of potential end users using the AI system output. * Indicates the studies included: (1) Dosovitsky et al [36]; (2) Benrimoh et al [37]; (3) Dimeff et al [38]; (4) Jacobs et al [39]; (5) Popescu et al [40]; (6) Prochaska et al [41]; (7) Prochaska et al [42]; (8) Deng et al [43]; (9) Garriga et al [44]; (10) Megerian et al [45]; and (12) Tanguay-Sela et al [46].

These 3 types of AI systems differ in relation to the intended end user’s engagement with the AI output to make a decision and with the extent of actionability of decisions the AI output is assisting (Figure 2 [31,36-46]). In Figure 2, *awareness* represents the extent to which the AI supports the end user’s understanding of the situation related to decision-making, while *acting on a decision* refers to more direct and explicit AI support on which decision to be made. It could be seen that the 3 systems have different interaction dynamics. The figure shows that different AI systems require varied engagement of end users, including patients and health care professionals, to finalize a decision. Moreover, the urgency and requirement to act on a health-related decision in a care process provided by the AI systems differ as well (Figure 2).

The dot’s locations represent the positions of the *intended primary end users* who are expected to interact directly with the AI system interface, and the intended primary decision-making utility (as represented in the 2 dimensions). The dot size reflects the number of AI systems that share the same location. The transparent background highlights the potential variety of involvement of secondary end users who may use the AI output alongside the primary intended user to make a decision.

In reference to Figure 2, the findings show diagnostic and predictive AI systems had the broadest potential number of primary end users from the health care and patients that needed to follow-up with the same AI output until reaching a decision without the need to interact with the AI interface. Treatment selection AI systems had a broader need for interaction through the same interface by both the health care professionals and the patients. The systems in self-help AI varied between supporting psychoeducation (closer to awareness) and psychotherapy (closer to acting on a therapeutic decision). Compared to the other types of AI systems, this was the most multifunctional type in relation to supporting various decisions.

#### Data Flow and User’s Interaction With the AI System

**Figure 3:**
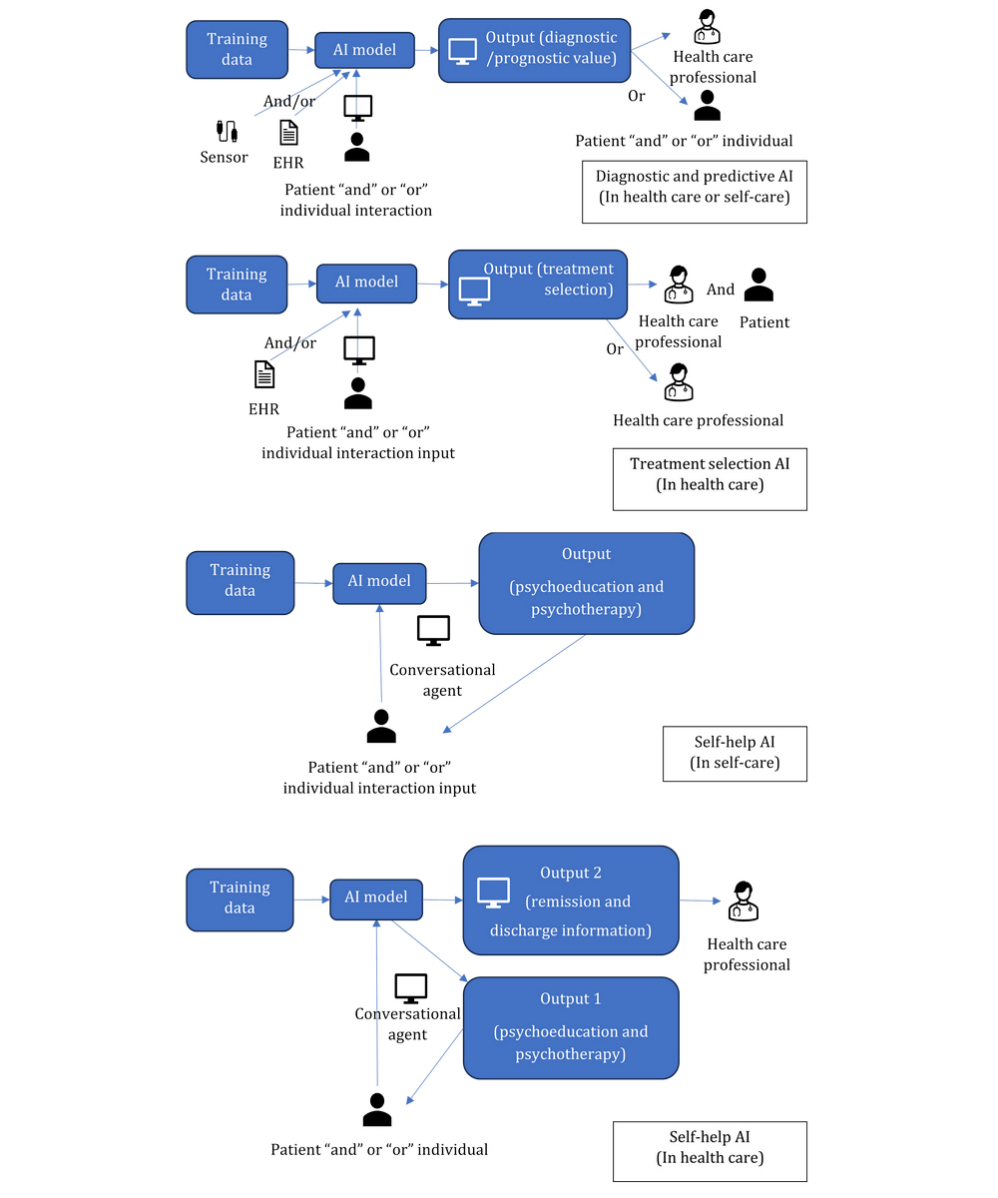
Illustration of the 4 data flow and interaction modalities of the 3 types of artificial intelligence (AI) systems. EHR: electronic health record.

It can be seen in a closer analysis of the use of AI systems for mental health issues that the 3 systems are different in the flow of data, both as input and output to the AI model; user interaction with the AI system; and the dynamics of the decision makers using the AI output. Four data flow and interaction modalities were found as seen in Figure 3.

Mapping the structure of the AI system’s use journeys shows 3 key differences: the variations in AI input data sources, the use of AI output interface, and the different user interaction interfaces along the AI use journey. First, the sources of AI input data varied in the 3 systems, and a broader range of sources of data were used in the diagnostic and predictive AI. This indicates a more diverse array of new input being incorporated into the AI data analysis. In contrast, self-help AI systems, which were based on conversational interactions, incorporated the end user’s textual interaction with a loop of self-help outputs. Second, the use of the AI output interface differed in the 3 AI systems, with more complicated dynamics of use in the treatment selection AI, where both patients and health care professionals potentially needed to interact with the same AI output interface. This is in contrast to the other 2 AI system types, where the interface was only used by 1 type of user at a time, either the health care professional or patient. Third, the interfaces used in the user journey from data input to output revealed variations in the dynamics of interaction. The interfaces for data input and output in the diagnostic and predictive AI and the treatment selection AI were separated, indicating a clear distinction between how information is entered into the system and how it is presented. In the self-help AI systems in health care settings, the input and output were integrated into a single interface for the patient’s or individual’s use, which in addition showed the potential for providing an extended interface for output for the health care professional to follow-up with the patient interaction.

### Evaluation of the Implementation and Use of the AI Systems to Support Decision-Making

#### Overview

The findings revealed a varied distribution in how the studies evaluated AI-based decision support systems. The human factor in the self-help AI had a predominant focus compared with the other 2 AI types. AI factors were more balanced in representation in diagnostic and predictive AI and treatment selection. The organization factor emerged as the least evaluated aspect in terms of frequency in all AI system types (Table 3). The assessment of the AI systems using the HOT-fit elements, as detailed in Table 3 and Multimedia Appendix 4 [33], reveals the different focus of evaluation across the 3 types of AI systems, illustrating potential challenges and enablers for use and implementation.

**Table 3:** Summary of the human, organization, and technology-fit (HOT-fit) elements found in the 3 types of artificial intelligence (AI) systems and key findings of potential challenges and enablers of use and implementation.

HOT-fit elements			AI system type		Key findings, challenges (–), and enablers (+)
**Human**					
	Amount and duration	Diagnostic and predictive, treatment selection, and self-help		(–) Prominent inconsistent duration of use found in self-help AI in self-care settings	
	Motivation to use	Self-help		(–) Potential lack of motivation for long-term of use without external support	
	Acceptance	Self-help		Different acceptance according to user’s socioeconomic status	
	Recurring use	Self-help		(–) Unstable recurring use can disturb the evaluation of self-care conversational agent’s effectiveness	
	Expectations and belief	Self-help		Most users expressed that the conversational agent met some of their goals or needs, only few users expressed that it met most or all their needs	
	Resistance and reluctance	Self-help		(–) Potential onboarding resistance and discontinuation after the first or second step in self-care settings	
	Percentage used	Treatment selection and self-help		(–) Low completion rates in self-help AI in self-care settings	
	Voluntaries of use	Self-help		Users are more likely to engage when initiated by the conversational agent	
	Knowledge and expertise	Treatment selection		(+) Knowledge about the AI model can potentially improve the ability to explain the AI output (–) Previous knowledge about the AI in general may reduce the trust	
	Overall satisfaction	Diagnostic and predictive and self-help		(+) Overall satisfaction was relatively high in relation to the amount of help the end users got from the system, the consistency of the system, and if they would use it again or recommend it for others	
	Perceived usefulness	Diagnostic and predictive, treatment selection, and self-help		(+) When evaluated most of participants perceived that the AI systems can successfully assist them	
	Satisfaction with specific functions	Treatment selection and self-help		Self-help AI satisfaction varied according to the self-help topic Treatment selection AI satisfaction was related to information the AI provided and can help with the communication with the patient	
	Decision making satisfaction	Treatment selection		(–) When evaluated only 60% expressed that the model is somehow satisfactory to support treatment decisions	
**Organization**					
	Communication	Treatment selection		(+) Potential participation in improving the communication of options or decisions	
	Clinical process	Diagnostic and predictive and treatment selection		(+) Positive responses on the ability to transform current clinical practice (–) Can lead to increase of workload by introducing additional steps into their practice	
	Localization	Diagnostic and predictive		Potential change of AI performance when used in different environments in relation to stability	
**Technology**					
	Ease of use	Diagnostic and predictive and self-help		(–) Potential difficulty requiring the need for technical support during the use of AI system	
	Technical support	Diagnostic and predictive		(–) Expressed need for assistance to get introduced to the system to be able to start using it	
	Usefulness of system features and functions	Diagnostic and predictive, treatment selection, and self-help		(+) Providing options, identification of cases and prioritization support features were found useful by participants of the different systems (–) The need for time to onboard to use the different features were expressed	
	Data accuracy	Diagnostic and predictive		(–) Outdated data and misrepresentation of data can lead to lower AI model accuracy	
	Trustworthiness	Treatment selection		(–) Affected by the end user’s knowledge, the AI processing methods, and onboarding use	
	Explainability	Diagnostic and predictive and treatment selection		(–) Lack of explainability can affect trust negatively	
	Usefulness	Diagnostic and predictive, treatment selection, and self-help		(–) Potential misalignment between what the end users found useful compared with AI intended purpose of use	
	Relevance	Diagnostic and predictive		(+) Most end users found the self-help service relevant to their wants.	
	Reliability	Treatment selection		(–) Potential misalignment of AI processing method compared with the health care method	
	Accuracy	Treatment selection		Potentially affected by the environment used in, and health care professional’s confidence in their decision.	
	Empathy	Treatment selection and self-help		(–) Potential skepticism by health care professionals “AI interprets data, but people are not data”	

In relation to the 3 types of AI systems, the study identified the following findings.

#### Diagnostic and Predictive AI

In relation to the evaluation of the use and implementation of diagnostic and predictive AI systems, the articles had a greater focus on evaluating technology elements compared with a less detailed evaluation of human and organization factors.

The human factor was considered only in terms of the amount and duration of use of the AI system, overall satisfaction, and perceived usefulness in the studies by Garriga et al [44] and Megerian et al [45] indicating positive outcomes in this regard.

In relation to the investigation of the technology factor, the main elements were accuracy and ease of use. The accuracy of the AI system was associated with several issues and considerations in relation to the relevance of the data used, illustrating the importance of including different stakeholders in the development of AI models to ensure that relevant data are included. However, in the study by Garriga et al [44] with a predictive AI system based on electronic health record data, the health care professionals highlighted that there was outdated information fed to the AI model and important qualitative data from the patients were not included. While in the study by Deng et al [43], the data used in the diagnostic AI model for children with autism were only based on the child’s input, excluding the input from their caregivers. The study illustrated that caregivers were concerned about not having their input for better outcomes.

The environment in which the AI system is used is found to contribute to affecting the accuracy. Deng et al [43] found that the prediction of the autism symptoms was less accurate when using the AI system in a less comfortable environment for the patients, and it gave more accurate output when implementing strategies that adjust the environment for the use of the AI system. Another consideration found affecting accuracy was health care professionals’ confidence in their own decisions. Megerian et al [45] showed that greater confidence in one’s decision in ruling out the disease over AI output was associated with less accuracy of negative diagnostics (compared with false positive accuracy, which was notably higher).

The ease-of-use element was evaluated in 2 studies reporting that the AI system was relatively easy to use, with the possible need for technical support due to use difficulties [43,44]. The design and ease of use of the AI system was found to influence the health care professional’s adoption of decisions provided by the AI system. The low level of understanding of the AI output by a few health care professionals in the study by Garriga et al [44] was a reason for them to not follow-up with the decisions of the AI system.

The evaluation of the organization factor was mainly focused on the clinical process element. Garriga et al [44] reported that the predictive AI tool was used as an additional resource to identify cases, and it led to additional workload due to the need for contacting more patients, arranging visits, and reviewing patient cases in a multidisciplinary team for medical SDM. While in terms of the clinical process change in this paper, the health care professionals reported that the tool did not change the method they followed to assess risk; it rather notified them of the unseen cases that might been missed in the standard practice processes. The identified facilitators for the adoption of the AI system in the study by Garriga et al [44] for the clinical processes were good training and technical support. In contrast, the barriers reported were related to concerns about the responsibility of acting on the AI output and the potential increase in workload and time when using the AI system.

#### Treatment Selection AI

Several elements affecting the use and implementation of AI systems were evaluated in this AI type within the 3 factors of the HOT-fit model.

Duration of use, users’ knowledge, and perceived usefulness were the main elements in the human factor found affecting the use of AI. The willingness of health care professionals to use the *treatment selection AI* systems was ≤5 minutes per session in the study by Benrimoh et al [37]. Moreover, 40% of the physicians in the study by Benrimoh et al [37] stated that they would only use the AI system in complicated or treatment-resistant patients, while 50% stated they would use it for all cases. The frequency and amount of use of the AI systems in the study by Jacobs et al [39] regarding *treatment selection AI* were associated with the amount of clinician’s knowledge in machine learning (ML). The increased familiarity of the health care professionals about ML was associated with the reduction of use of the ML recommendation, despite the higher level of perceived utility of ML. The health care professionals, in the 4 studies looking at the contexts in which treatment selection AI was used, expressed two main ways in which they found the AI system useful: (1) improving patient-clinician communication, which included improving patient understanding for treatment options, making communication less personal, and improving trust in treatment and (2) improving clinical practice such as potentially saving time, providing more objectivity to the practice, confirming or suggesting options, using it as a guideline, or as a source of extra information.

The main technology-related elements in this type of AI system were related to accuracy and trustworthiness. Consistent with the findings in the diagnostic and preventive AI system, the increased familiarity of ML was associated with a decrease in accuracy, despite the increase in clinician confidence in their decisions [39]. Three studies [37,40,46] investigated trustworthiness of the AI system and most of the participants had a high level of trust in using the AI system. Trust in the AI model was reported in the study by Tanguay-Sela et al [46] to be improved with sessions only for participants who reported their trust as unsure in the first session of use, while ratings of either low or high levels of trust did not change for participants who reported low or high levels of trust in the first session (ie, a low level of trust did not improve with increased sessions of use). A lack of trust in particular AI features were found to be an aspect affecting the overall trust of the AI system. The participants in the study by Tanguay-Sela et al [46] reported that their trust in the AI system was reduced due to them finding one of the features of the AI analysis irrelevant. A lack of explainability and evidence of the used models were mentioned in the study by Tanguay-Sela et al [46] as potentially affecting trust in the AI system.

AI system use in terms of the organization factor has been found to affect clinical processes, particularly in patient-physician communication. Some health care professionals in the study by Tanguay-Sela et al [46] had negative opinions about the use of the AI tool stating that it was negatively interfering with the interaction flow with the patient, while others saw it as a valuable tool in the communication process. Benrimoh et al [37] showed that physicians turned their screen to include the patients in the medication selection process even though it was not required in the study design. The AI system was reported in the study by Tanguay-Sela et al [46] to be potentially used in clinical processes other than direct treatment selection for the health care professionals. It was mentioned that the AI system can support clinical processes such as in assisting communication and SDM by visualizing the different options and being able to motivate options for the patients. For this to be valid, the study illustrated that the AI tool needs to be well understood by the physicians, and with transparent explanation of the options, for them to be able to communicate it to the patients.

#### Self-Help AI

The human factor was the center of focus of evaluation in this type of AI system. Duration of use, percentage used, frequency of use, acceptability, and user satisfaction were the main elements evaluated.

More consistent duration of use and better completion rates of self-help content were found in the conversational agents with health care–supervised type [38], compared with the unguided self-care AI agents [36,41,42]. The duration of use in the study by Dosovitsky et al [36] was inconsistent ranging from 31 minutes to 48 hours to finish 1 module of content. The frequency of use in the studies by Dosovitsky et al [36] and Prochaska et al [42] was inconsistent as well, with a connection found in relation to the intrinsic motivation and passive engagement of the users with the conversational agents [36], where it was found that the users would be more likely to engage in a session when it is initiated by the conversational agent. Satisfaction scored relatively high in all studies looking at satisfaction and perceived usefulness, but in relation to acceptability of use for self-care conversational agents it was found to vary according to the severity of the mental health condition, the socioeconomic status, such as cultural or racial background, marital status, and education [41].

Technology elements such as ease of use, usefulness, and empathy were investigated only in score-based evaluation showing positive outcomes, while organization elements in this AI system type were not found to be investigated.

## Discussion

### Principal Findings

This study explored the empirical evidence surrounding the use and implementation of AI systems for decision-making support in mental health. The identified studies primarily focused on AI systems developed for health care and self-care settings, with articles predominantly sourced from high-income countries. The state of research on AI systems in mental health is in the preimplementation stage in clinical processes. Although studies have evaluated the use of AI systems and their potential impact on clinical processes, none of the studies have explored the full adoption or long-term implementation of AI systems in care settings. Of the 12 studies, 8 (67%) evaluated the use of AI systems in real-time care use, while 4 (33%) studies focused on simulations or clinical case studies.

A total of 3 main types of AI systems were categorized according to their utility in mental health: diagnostics and predictive AI, treatment selection AI, and self-help AI. These systems exhibited differences in their use process and interaction with end users. The findings about the types of AI systems and the variety of end users engaged in using these systems were found to be consistent with previous research [47-49], which underscored the potential use of AI in mental health, and emphasized the importance of understanding the sustainable design, which enables the collaboration in decision-making and the integration of these technologies into clinical processes. The AI systems provided support to a diverse range of actors both in health care, including primary care physicians, community psychiatric nurses, psychiatrists, and occupational therapists, as well as outside the health care services, including patients, individuals with mental health problems, and caregivers.

Evaluating the AI systems through the lens of HOT-fit highlighted several challenges in the integration and use of AI systems in care flow. These challenges included potential issues affecting accuracy and use biases, increased demand, physician-patient communication, and engagement with the AI system in self-care. However, despite the existing insights, there remains a pronounced lack of evidence that can help assess the integrative use and implementation of AI systems in different care contexts.

### AI System Design for Decision Support and Interaction

The studies demonstrated the diverse interaction dynamics between end users of AI decision-making support systems. Interactions were influenced by the types of users and the data flow within the AI systems. There were variations in how data flows through the 3 types of AI systems. From the methods used to input data, to the types of interfaces presented to users, to the inner functionality of the AI models, and finally, to the nature of the output data—each system painted a unique picture. These variations align with the 4 central human-computer interaction (HCI) aspects identified by Rundo et al [50] for investigating decision-support systems, emphasizing the necessity of integrating HCI principles into AI technology development and implementation to ensure they effectively serve user needs, enhance decision-making outcomes, and ensure the sustainable use of AI in decision-making in mental health care.

Diagnostic and predictive AI systems in the articles showed the greatest diversity in data sources, which can complicate user understanding and create a *black-box* effect due to lack of explainability, which can reduce user acceptance and decision-making quality [51,52]. Furthermore, how the AI model functions to support decision-making based on data input is crucial for acceptance and sustainable use [12,53]. Nevertheless, none of the studies showed how the AI model is being sustainably trained or how the end user’s use of the AI systems contributed to the continual learning process of the AI model. This process is a fundamental part of AI or ML apps that provide continual adaptation of the AI model for improvement of its performance with further use [54]. End user–inclusive strategies, such as the human-in-the-loop approach, where health care professionals and patients actively participate in providing feedback to enhance the AI model’s functionality, can be useful in addressing this knowledge gap. This ensures that both humans and technology are aligned toward common long-term goals, promoting their joint improvements and effectiveness [54,55].

Three main types of interfaces for end users were discernible when examining the use and data flow within the AI systems: data collection interfaces, AI output visualization interfaces, and combined input and output interfaces in which interaction loops take place as in self-help AI type. These interfaces were designed to be used by a single user (patient or health care professional) or multiple end users. The differences in interface types and end-user interaction can pose challenges in examining these systems and ensuring fluency in care flows. For example, while self-help AI interaction loop interfaces can simplify the flow of use and enhance automation, compared with separated input and output interfaces, it can also complicate measuring interaction effectiveness in the health care [56-58]. Moreover, when multiple end users with different backgrounds use a single AI interface for decision-making, there is a growing need for interfaces to provide on-demand customized explanations to fit the understanding and reasoning of multiple stakeholders [59]. This also points to the need for co-design approaches to involve stakeholders from the early stages of development [60-62].

Using AI system interfaces to support decision-making presents varied dynamics of interaction, not only between the end users and the AI system but also among the end users themselves. Interestingly, none of the reviewed studies included an AI system explicitly designed to facilitate SDM between health care professionals and patients nor did they assess the role of SDM when using AI systems. This absence raises questions about the extent to which collaboration between health care professionals and patients, often highlighted as crucial for improving decision quality in mental health care, is being integrated into AI-assisted processes [51,52,63]. Drake et al [64] emphasized the need to incorporate SDM into the clinical workflows for effective implementation of patients’ involvement [64]. As AI systems become more integrated into clinical decision-making workflows, they could alter traditional clinician-patient interactions, introducing new modalities such as clinician-AI, clinician-patient, and patient-AI interactions [65]. Each of these modalities may bring normative challenges that affect the roles of agency, transparency, trustworthiness, and responsibility among end users. Future research should explore how to thoughtfully integrate SDM in the design, implementation, and practical use of AI systems in care processes, while also addressing the broader normative shifts that may arise in clinical workflows because of AI integration.

### Types of AI Systems for Supporting Decision-Making

The evaluation of the 3 types of AI systems described in the articles using the HOT-fit framework highlighted several challenges impacting the use and potential implementation of these systems in mental health care processes. General challenges were observed affecting the accuracy of the AI systems due to the use of nonrepresentative or skewed data input, in addition to the selective use of the AI system participating in potential future biases. Other challenges found more in one AI system than another included the increase in usual care process steps when using the predictive AI systems, the need for adjustment for patient-physician communication when using the treatment selection AI type, and user engagement issues when using the conversational agents in self-help AI.

It was noted in some studies that health care professionals had a selective use of the AI systems for cases with specific severity, or the systems were preferred to be used by specific health care professionals more than others. This selective use with the continuous learning of the AI model may lead to feedback loop biases. This type of bias refers to the distortion of the AI output affected by the skewed use for specific cases over an extended span of time. Consequently, the AI output becomes more accurate to the selected cases and less toward the general use, which can affect the overall accuracy [66]. Furthermore, users in some of the studies described that the AI systems are useful for purposes that deviate from the AI model’s primary objective. If the potential deviated use was not considered during the implementation efforts, this issue can lead to *concept drift* bias. This refers to the bias that may happen when the AI processing method differs from the actual use of the AI system leading to degradation of the AI output performance over time [67]. This underscores the need for including health care professionals and patients in the development and implementation phases to ensure that the AI system is designed and implemented in alignment with the sustainable use dynamics [68,69]. Co-design methods for analyzing sociotechnical scenarios can facilitate this by engaging stakeholders in early phases using prototype drafts to validate key AI-related concepts such as its goal and potential risks [62]. Early stakeholder involvement is also essential for building trust and ensuring implementation and adoption. Research shows that collaboration among a diverse, interdisciplinary team, including clinicians, potential AI users, hospital leaders, quality improvement teams, human resources, and IT departments is key to achieving effective implementation. This collective expertise ensures that the AI system meets clinical needs, aligns with organizational goals, integrates smoothly into workflows, and addresses technical, operational, and user concerns [70].

In diagnostic and predictive AI, the utility of the AI systems in detecting mental health state and identifying potentially hidden cases can disrupt common clinical processes by increasing demand and requiring additional steps or time to validate the decision support presented by the AI system. Nevertheless, none of the studies evaluated how this disruption of the care process can be adjusted to and integrated into the usual care routines. If not addressed in the future, this can lead to a dilemma of consequences between undertrusting or overtrusting the AI output in decision-making. Undertrusting of the AI output may require additional steps to validate the decisions provided by the AI system leading to additional workload and time consumption or abandoning the AI system use [12,71]. In contrast, the overreliance on AI systems, particularly when they have a high success rate, can lead to *automation bias*, where decision makers favor AI recommendations over other sources information including their own [72]. This may result in increased dependency on AI, discarding both health care professionals’ and patients’ judgments and preferences. Such a shift could move clinical care away from person-centered care toward the paternalized style of decision-making raising ethical and safety challenges [73,74]. A prominent example is a commercial AI model implemented in the health care system in the United States, which led to a racial bias discriminating access to health care for millions of patients [75]. These concerns highlight the need for caution in scaling and adopting AI for critical decision-making tasks without robust evidence. Initiatives such as the AI Act aim to address these ethical issues by setting requirements for AI development and implementation. Current recommendations emphasize that final decisions in diagnosis and care should remain with human judgment, with AI serving as an assistive tool, a view supported by numerous studies in mental health care [8,76-78].

Treatment selection AI systems were the only type evaluating the patient-physician relationship when using the AI system. Health care professionals used the AI system in their communication with the patients in some of the studies to discuss options and guidelines. However, this integration of AI decision support can disrupt the communication process, introducing sociorelational challenges into the clinical processes that need to be addressed in the design and implementation of the AI to avoid such challenges [13]. The average time willingness to use the AI system was limited to 5 minutes in one of the studies. This time constraint may present a challenge of integrating the AI support into the patient-physician communication. It highlights the need for efficient and explainable AI that can facilitate communication within a short span of time and tailored to the individualized care processes considering both technical and end-user preferences [79].

A main challenge identified in the studies with self-help AI systems based on conversational agents is inconsistency in user engagement, including frequency of use, duration, and completion rates. The self-help AI systems, primarily used for psychotherapy and psychoeducation, face distinct challenges in both self-care and health care settings. Low engagement can reduce the effectiveness of self-help AI systems, particularly because behavioral change and cognitive behavioral therapy (CBT) techniques require long-term engagement to attain effectiveness [80,81]. While conversational agents show great potential in facilitating mental health services [82,83], several studies indicate a lack of clarity about effectiveness and evidence-based design for therapy and mental health assessment [84-88]. This challenge highlights the need for additional research on long-term effectiveness and reliability of conversational agents across various mental health applications. Jabir et al [89] examined how conversational agents are typically evaluated and found that effectiveness assessment usually includes at least 1 clinical outcome and 1 user experience outcome. Given the need for sustained engagement with CBT-based conversational agents and the findings of our study, we recommend that future studies incorporate a third type of outcome measurement to better validate effectiveness. This additional measurement should focus on behavioral change, which is a fundamental aspect of CBT and chronic health conditions [90,91]. Cole-Lewis et al [92] demonstrated that engagement with technology can lead to real-world behavior changes and consequently supporting health outcomes. Including behavioral change as an outcome is important to ensure that the mediated behaviors are successfully targeted not just the clinical outcomes or the user engagement. This is crucial because long-term life changes may mislead the association between self-help AI effectiveness and other positive life-related factors. Using behavior change techniques can serve as validated instruments for assessing health or decision-making behavior change, thus guiding the evaluation of conversational agents [93-97].

Overall, from the findings it is evident that successful integration and implementation of AI systems in existing mental health care workflows requires substantial efforts to create evidence regarding its sustainable use. There is a lack of empirical studies evaluating the long-term implementation of AI in mental health care. Nair et al [70] identified strategies and barriers for successful implementation that lead to sustainable use in 3 essential areas (planning, implementing, and sustaining the use). When integrating AI systems for decision-making support, the evaluation of changes in clinical workflows needs to be considered proactively during the planning phase to avoid unseen organizational or increased demand issues when put in place [68]. This step requires the inclusion of multidisciplinary individuals who will engage with the workflow using simulations or prototypes to enable the detection of any barriers or potential risks before putting the integrated AI systems in place [98,99]. Regarding implementation strategies and ensuring sustainable use, it is important to investigate approaches relevant to the context of mental health care processes addressing resistance of change, staff training, and monitoring of AI performance. This can help create evidence participating in planning for sustainable AI systems. Future studies, including case studies and longitudinal implementation research, are necessary to generate evidence for how to successfully plan, implement, and integrate AI for sustainable use in mental health. These studies can support proposing strategies for integrating AI decision-making utility in mental health practices, with the consideration to, including all relevant stakeholders in the research process.

### Strengths and Limitations

Some methodological considerations are worth noting. The literature search was conducted using 5 relevant databases, which were selected based on the study’s focus and the research field. However, it is important to acknowledge that using different databases may yield varying results, potentially affecting the comprehensiveness of the review. The keywords used in the search strategy were thoroughly discussed within the research group and with librarian assistance to align with the study’s aims. Nonetheless, the evolving nature of interdisciplinary field, such as health care, implementation science, and AI technology means that the literature may use a broad range of terminology. This variability could impact the identification and inclusion of relevant studies, as different keywords might yield different sets of literature.

Furthermore, the decision to include only AI systems with technology readiness level ≥6 intended to focus the exploration on AI systems that are close to the implementation phase and investigating the AI use in care settings. While this criterion ensured relevance to real-world applications, it also narrowed the scope of the review, excluding studies that focus on the earlier developmental stages of AI models. This limitation means that the review does not address the potential capabilities of emerging AI systems that are still in the research or proof of concept phase but might have profound implications for mental health applications in the future. In addition, the limited number of studies available in implementation phase restricts the ability to draw generalized conclusions about the long-term effectiveness or scalability of these AI systems in mental health.

Moreover, 2 researchers independently conducted the selection and review, enhancing the reliability and reducing potential bias in study inclusion. However, assessing SDM in relation to these AI systems posed a challenge, as most studies did not report on the interaction dynamics between health care professionals, patients, and the AI system in a mutual decision-making context. This lack of detailed reporting on SDM limits the understanding of how AI influences or facilitates collaborative care in mental health.

### Implications and Future Research

This study sheds light on the knowledge gaps regarding the empirical evidence of the use and integration of AI systems in mental health decision-making processes, offering insights that can guide the development and design of human-centered AI systems. It underscores the need to consider the utility of AI systems and end-user perspectives in the planning for the development and implementation of AI systems to support decision-making in mental health, in addition to the need for more empirical evidence to validate the factors affecting behavioral sustainable use. Furthermore, this study presents the current state of complexity in which the HCI perspective is fundamental in combination with co-design methodologies to ensure person-centered care when implementing AI systems in care processes.

Future research is advised to explore the integration of support for SDM in the AI system’s design and development, addressing the gap identified in the current literature. In addition, there is a need for conceptual refinement of the current SDM process due to the disruptive effect of AI use. As the care process is evolving from the traditional physician-patient SDM, potentially adding the AI component as a third decision maker in the SDM dynamics. This adjustment is leading to changes in the essential elements of the process, and the 3-talk model, in particular when it comes to the technology-human SDM dynamics [28,100]. Conducting implementation studies are needed for creating robust empirical evidence, and to address potential long-term challenges by identifying barriers and facilitators of AI implementation and sustainable use in mental health care.

### Conclusions

The scoping review demonstrated the diverse range of uses of AI in the mental health field, including health care settings, self-care contexts, and hybrid approaches. However, there was a lack of empirical evidence from an implementation perspective on how the AI systems should be integrated into clinical processes. None of the studies discussed how to adjust clinical processes to accommodate aspects such as patient-physician communication and SDM.

The results showed that the utility of AI systems in supporting decision-making in mental health varies and illustrates the complexity of evaluating the utility of decision-making, influenced by the type of actors involved in decisions, the level of decision actionability, and the dynamics of data flow through the AI system. The evaluation of the use of AI systems in care settings through human, organization, and technology lenses revealed several challenges for the implementation of AI systems for sustainable use in care settings. As a consequence, these use- and implementation-related challenges may impact the accuracy of the decisions supported by the AI system, disrupt physician-patient communication, pose trust issues, and present engagement problems impacting the effectiveness and adoption of AI decision support. Future studies are needed to address stakeholder’s needs in AI systems design, evaluate the implementation of AI systems in clinical processes for sustainable use, and assess the integration of SDM in AI systems.

## Appendix

Multimedia Appendix 1 PRISMA-ScR (Preferred Reporting Items for Systematic Reviews and Meta-Analyses Extension for Scoping Reviews) checklist.

Multimedia Appendix 2 Search strategy.

Multimedia Appendix 3 Artificial intelligence systems characteristics.

Multimedia Appendix 4 All elements used in the human, organization, and technology-fit framework with reference to the articles.

## Abbreviations

AI: artificial intelligence
HCI: human-computer interaction
HOT-fit: human, organization, and technology-fit
PRISMA: Preferred Reporting Items for Systematic Reviews and Meta-Analyses
PRISMA-ScR: Preferred Reporting Items for Systematic Reviews and Meta-Analyses Extension for Scoping Reviews
RQ: research question
SDM: shared decision-making
