# Supplementary material for: The Use of AI in Mental Health Services to Support Decision-Making: Scoping Review
Source: J Med Internet Res. 2025 Jan 24;27:e63548. doi: 10.2196/63548 (PMC11806275; doi:10.2196/63548)
Supplement: Multimedia Appendix 2 [file jmir_v27i1e63548_app2.doc]

## **Multimedia Appendix 2.** Search strategy

Table S1. Search results from the five databases.

| Database /Date searched | PubMed | Scopus | PsycINFO -ProQuest | Web of Science core collection | CINAHL-Ebsco | Sum |
| --- | --- | --- | --- | --- | --- | --- |
| Number of articles found from the searches | 557 | 576 | 180 | 379 | 81 | 1773 |
| Number of duplicates |  | | | | | 556 |
| Total number minus duplicates |  | | | | | 1217 |

Table S2. Searching keywords and queries used in the databases.

| Database | Search keywords and queries |
| --- | --- |
| PubMed | ("mental health" [mh] OR "mental illness" OR "psychological distress" [mh] OR "behavioral symptoms" [mh] OR "stress" OR "anxiety" OR "depression" OR psychiatr* OR "mental disorders" [mh] OR suicid* OR "adhd" OR "autism" OR "bipolar disorder" OR "depressive disorder" OR "post traumatic" OR "obsessive compulsive disorder" OR "compulsive behavior" OR "schizophrenia") AND ("artificial Intelligence" [mh] OR "neural networks" OR "deep learning" OR "machine learning" OR "computational intelligence" OR "supervised machine learning" OR Robot*)) AND ("decision making, computer-assisted" [mh] OR "decision support systems, clinical" [mh] OR "decision Support techniques" [mh] OR recommend* OR "decision support")) AND (implement* OR innovat* OR improv* OR "deploy" OR "deliver" OR "launch") |
| Scopus | (TITLE-ABS-KEY("mental health" OR "mental illness" OR "psychological distress" OR "behavioral symptoms" OR "stress" OR "anxiety" OR "depression" OR psychiatr* OR "mental disorders" OR suicid* OR "adhd" OR "autism" OR "bipolar disorder" OR "depressive disorder" OR "post traumatic" OR "obsessive compulsive disorder" OR "compulsive behavior" OR "schizophrenia") AND TITLE-ABS-KEY ( "artificial Intelligence" OR "neural networks" OR "deep learning" OR "machine learning" OR "computational Intelligence" OR "supervised machine learning" OR robot*) AND TITLE-ABS-KEY ("decision making" OR recommend* OR "decision support") AND TITLE-ABS-KEY (implement* OR innovat* OR improv* OR "deploy" OR "deliver" OR "launch") |
| PsycINFO (ProQuest) | ("mental health" OR "mental illness" OR "psychological distress" OR "behavioral symptoms" OR "stress" OR "anxiety" OR "depression" OR psychiatr* OR "mental disorders" OR suicid* OR "adhd" OR "autism" OR "bipolar disorder" OR "depressive disorder" OR "post traumatic" OR "obsessive compulsive disorder" OR "compulsive behavior" OR "schizophrenia") AND ("artificial Intelligence" OR "neural networks" OR "deep learning" OR "machine learning" OR "computational intelligence" OR "supervised machine learning" OR Robot*) AND ("decision making" OR "clinical decision support systems" OR "decision support techniques" OR recommend* OR "decision support") AND (implement* OR innovat* OR improv* OR "deploy" OR "deliver" OR "launch") |
| Web of Science Core Collection | (TS=("mental health" OR "mental illness" OR "psychological distress" OR "behavioral symptoms" OR "stress" OR "anxiety" OR "depression" OR psychiatr* OR "mental disorders" OR suicid* OR "adhd" OR "autism" OR "bipolar disorder" OR "depressive disorder" OR "post traumatic" OR "obsessive compulsive disorder" OR "compulsive behavior" OR "schizophrenia")) AND TS=("artificial Intelligence" OR "neural networks" OR "deep learning" OR "machine learning" OR "computational intelligence" OR "supervised machine learning" OR robot*)) AND TS=("decision making" OR recommend* OR "decision support" )) AND TS=(implement* OR innovat* OR improv* OR "deploy" OR "deliver" OR "launch")) |
| CINAHL (Ebsco) | ("mental health" OR "mental illness" OR "psychological distress" OR "behavioral symptoms" OR "stress" OR "anxiety" OR "depression" OR psychiatr* OR "mental disorders" OR suicid* OR "adhd" OR "autism" OR "bipolar disorder" OR "depressive disorder" OR "post traumatic" OR "obsessive compulsive disorder" OR "compulsive behavior" OR "schizophrenia") AND ("artificial Intelligence" OR "neural networks" OR "deep learning" OR "machine learning" OR "computational intelligence" OR "supervised machine learning" OR robot*) AND ("decision making" OR recommend* OR "decision support") AND (implement* OR innovat* OR improv* OR "deploy" OR "deliver" OR "launch") |
