# Supplementary material for: The Use of AI in Mental Health Services to Support Decision-Making: Scoping Review
Source: J Med Internet Res. 2025 Jan 24;27:e63548. doi: 10.2196/63548 (PMC11806275; doi:10.2196/63548)
Supplement: Multimedia Appendix 3 [file jmir_v27i1e63548_app3.docx]

Multimedia Appendix 3 - AI systems characteristics

| Reference | Technology | Mental health and decision support | Types of AI system |
| --- | --- | --- | --- |

|  | AI model type | AI system interface described | AI input data as described in the articles | Actors (Users) | Mental health process | Decisions the AI supporting |  |
| --- | --- | --- | --- | --- | --- | --- | --- |

| #1 [1] | Type not specified (using AI or AI model) | Conversational agent “TESS” used through Facebook messenger | **(1) Trained** - 12 modules of depression psychoeducation and CBT content.  **(2) System use input** - Interaction with the CA. | Patients and individuals seeking self-care | Psychoeducation and self-help | Self-help decisions, and awareness of treatment options | Self-help AI |
| --- | --- | --- | --- | --- | --- | --- | --- |
| #2 [2] | Deep-learning model | Digital platform “Aifred” used through mobile application or personal computer | **(1) Trained** - Clinical and demographic datasets    **(2) System use input** - Patient standardized questionnaires. | Health professionals  (1) Psychiatrists  (2) Family medicine | Treatment | Medication selection | Treatment selection AI (recommendations) |
| #3 [3] | Type not specified (using AI or AI model) | Conversational agent “Jasper Health” within a tablet-based application  With multiple interface  (1) Patient interface  (2) Physican decision-support interface | **(1) Trained** - Four evidence-based practices of Suicide including psycho-education and DBT content.  **(2) System use input**  - Self-report questionnaires (suicide risk assessment). - Interaction with the CA. | A. Patients visiting emergency care  B. Health professionals  (1) Psychiatrist  (2) Psychiatric nurse practitioner | Planning, treatment, and case management | (1) Healthcare professionals:  Discharge/Disposition  Patients:  (1) Prepare them for the ED (2) Psychoeducation for surviving after returning home | Self-help AI |
| #4 [4] | Machine learning model | Interface with ML recommendations with explanation (without further description) | **(1) Trained** - Medical record data  **(2) System use input**  - No use input was described. | Healthcare professionals working in  (1) Primary care  (2) Psychiatry | Treatment | Medication selection | Treatment selection AI (recommendations) |
| #5 [5] | Deep-learning model | Digital platform “Aifred” used through mobile application or personal computer | **(1) Trained** - Clinical and demographic datasets    **(2) System use input** - Patient standardized questionnaires. | Healthcare professionals  (1) Family physicians  (2) Psychiatrists | Treatment | Medication selection | Treatment selection AI (recommendations) |
| #6 [6] | Type not specified (using AI or AI model) | Conversation al agent “Woebot-SUDs” through a mobile application | **(1) Trained** Content of substance misuse psychoeducation and CBT.  **(2) System use input**  - Interaction with CA.  - Mood tracking input. | Patients and individuals seeking self-care | Psychoeducation and self-help | Self-help decisions, and awareness of treatment options | Self-help AI |
| #7 [7] | Type not specified (using AI or AI model) | Conversational agent “Woebot-SUDs” through a mobile application | **(1) Trained**  Content of substance misuse psychoeducation and CBT.  **(2) System use input**  - Interaction with CA.  - Mood tracking input. | Patients and individuals seeking self-care | Psychoeducation and self-help | Self-help decisions, and awareness of treatment options | Self-help AI |
| #8 [8] | Machine-learning models | Sensory management recommendation system used through iOS mobile application and smartwatch application | **(1) Trained**  - Clinical data  **(2) System use input**  - Real-time psychological data  - Real-time surrounding audio data | Patients and individuals seeking self-care  (1) Children with autism.  (2) Their caregivers.  (3) Teachers. | Identification and monitoring of symptoms | Self-help decisions | Diagnostics and predictive AI (Severity based) |
| #9 [9] | Machine learning model | In the hospital clinical dashboard using the EHR of the hospital’s patients | **(1) Trained**  - Real-world EHR data.  **(2) System use input**  - No data | Healthcare professionals (1) Doctors  (2) Occupational therapists  (3) Duty workers  (4) Social workers  (5) Community psychiatric nurses  (6) Psychiatrist  (7) Team managers | Prevention | Identification of risk of mental health crisis cases | Diagnostics and predictive AI |
| #10 [10] | Machine-learning model | **With multi-interface**  (1) Mobile application interface for the patients and their caregivers.  (2) Healthcare portal through a browser. | **(1) Trained**  Not clear  **(2) System use inputs**  - Caregiver questionnaire  - Caregiver video  - Healthcare provider questionnaire | A. Healthcare professionals  (1) Primary care physicians  B. Patients and individuals seeking self-help  (1) Children with autism.  (2) Their caregivers. | Diagnosis | Providing positive or negative diagnostic value of an autism case | Diagnostics and predictive AI (Positive/Negative) |
| #11 [11] | (1) Machine-learning model  (2) Natural language processing; | Therapy-specific platform (Eleos Health) | **(1) Trained**  Not clear  **(2) System use**  - Audio data from treatment conversations (e.g.: behavioral therapy) | Healthcare professionals  (1) Psychologists  (2) Social workers  (3) Licensed counselors | Treatment | Supporting psychotherapy sessions (Behavioral therapy) | Was not included due to the lack of information about how it was used in the study. |
| #12 [12] | Deep-learning model | Digital platform “Aifred” is used through mobile applications or personal computer | **(1) Trained** - Clinical and demographic datasets    **(2) System use** - Patient standardized questionnaires. | Healthcare professionals  (1) Primary care physician  (2) Psychiatrist | Treatment | Medication selection | Treatment selection AI (recommendations) |

**References**

1. Dosovitsky G, Pineda BS, Jacobson NC, Chang C, Bunge EL. Artificial intelligence chatbot for depression: descriptive study of usage. JMIR Formative Research. 2020 Nov 13;4(11):e17065. PMID: 33185563. doi: 10.2196/17065.

2. Benrimoh D, Tanguay-Sela M, Perlman K, Israel S, Mehltretter J, Armstrong C, et al. Using a simulation centre to evaluate preliminary acceptability and impact of an artificial intelligence-powered clinical decision support system for depression treatment on the physician–patient interaction. BJPsych open. 2021 Jan 6;7(1):e22. PMID: 33403948. doi: 10.1192/bjo.2020.127.

3. Dimeff LA, Jobes DA, Koerner K, Kako N, Jerome T, Kelley-Brimer A, et al. Using a tablet-based app to deliver evidence-based practices for suicidal patients in the emergency department: pilot randomized controlled trial. JMIR mental health. 2021 Mar 1;8(3):e23022. PMID: 33646129. doi: 10.2196/23022.

4. Jacobs M, Pradier MF, McCoy Jr TH, Perlis RH, Doshi-Velez F, Gajos KZ. How machine-learning recommendations influence clinician treatment selections: the example of antidepressant selection. Translational psychiatry. 2021;11(1):108. doi: 10.1038/s41398-021-01224-x.

5. Popescu C, Golden G, Benrimoh D, Tanguay-Sela M, Slowey D, Lundrigan E, et al. Evaluating the clinical feasibility of an artificial intelligence–powered, web-based clinical decision support system for the treatment of depression in adults: longitudinal feasibility study. JMIR formative research. 2021 Oct 25;5(10):e31862. PMID: 34694234. doi: 10.2196/31862.

6. Prochaska JJ, Vogel EA, Chieng A, Baiocchi M, Maglalang DD, Pajarito S, et al. A randomized controlled trial of a therapeutic relational agent for reducing substance misuse during the COVID-19 pandemic. Drug and Alcohol Dependence. 2021 Oct 1;227:108986. PMID: 34507061. doi: 10.1016/j.drugalcdep.2021.108986.

7. Prochaska JJ, Vogel EA, Chieng A, Kendra M, Baiocchi M, Pajarito S, et al. A therapeutic relational agent for reducing problematic substance use (Woebot): development and usability study. Journal of medical Internet research. 2021 Mar 23;23(3):e24850. PMID: 33755028. doi: 10.2196/24850.

8. Deng L, Rattadilok P. A sensor and machine learning-based sensory management recommendation system for children with autism spectrum disorders. Sensors. 2022 Aug 3;22(15):5803. PMID: 35957356. doi: 10.3390/s22155803.

9. Garriga R, Mas J, Abraha S, Nolan J, Harrison O, Tadros G, et al. Machine learning model to predict mental health crises from electronic health records. Nature medicine. 2022 Jun;28(6):1240-8. PMID: 35577964. doi: 10.1038/s41591-022-01811-5.

10. Megerian J, Dey S, Melmed R, Coury D, Lerner M, Nicholls C, et al. Evaluation of an artificial intelligence-based medical device for diagnosis of autism spectrum disorder. NPJ Digit Med. 2022. 2022. doi: 10.1038/s41746-022-00598-6.

11. Sadeh-Sharvit S, Rego SA, Jefroykin S, Peretz G, Kupershmidt T. A Comparison Between Clinical Guidelines and Real-World Treatment Data in Examining the Use of Session Summaries: Retrospective Study. JMIR Formative Research. 2022;6(8):e39846. doi: 10.2196/39846.

12. Tanguay-Sela M, Benrimoh D, Popescu C, Perez T, Rollins C, Snook E, et al. Evaluating the perceived utility of an artificial intelligence-powered clinical decision support system for depression treatment using a simulation center. Psychiatry Research. 2022 Feb;308:114336. PMID: 34953204. doi: 10.1016/j.psychres.2021.114336.
