# Supplementary material for: The Use of AI in Mental Health Services to Support Decision-Making: Scoping Review
Source: J Med Internet Res. 2025 Jan 24;27:e63548. doi: 10.2196/63548 (PMC11806275; doi:10.2196/63548)
Supplement: Multimedia Appendix 4 [file jmir_v27i1e63548_app4.docx]

Multimedia Appendix 4 – All elements used in the HOT-fit framework with reference to the articles. The variables used in the framework adapted from [1]

| Human | | Organization | | Technology | | |
| --- | --- | --- | --- | --- | --- | --- |
| System use | User satisfaction | Structure | Environment | System quality | Information quality | Service quality |
| Amount/duration(4t,5t,1s,3s, 6s,7s)  Motivation to use (1s)  Knowledge/expertise (4t,12t)  Acceptance (6s,7s)  Percentage used (5t, 1s)  Recurring use (9d, 10d,6s,7s)  Expectations/belief (7s)  Resistance/reluctance (1s)  Voluntaries of use (1s)  Use by whom? (direct vs. chauffeured use,) actual vs. reported us  Nature of use (use for intended purpose appropriate use, type of information used)  Purpose of use, level of use (general vs. specific) report acceptance  Attitude  Training | Overall satisfaction (9d,3s,6s,7s)  Satisfaction with specific functions (2t,6s)  Perceived usefulness (9d,2t,4t,7s)  Enjoyment  Software satisfaction  Decision making  satisfaction (12t) | Clinical process (9d,12t)  Communication (2t,12t)  Nature (type, size) culture  planning  Strategy  management  Autonomy  Leadership  Top management Support  medical sponsorship  champion  mediator  Teamwork | Localization (8d)  Financing source  Government  politics  Competition  Inter-organizational relationship  population served  external communication | Ease of use (8d,9d,3s)  Technical support (8d)  Usefulness of system features and functions (9d,12t,6s)  Data accuracy (9d)  Data currency  Database contents, , ease of learning, availability, , flexibility, reliability, security, efficiency, resource utilization, response time, turnaround time | ^1^Trustworthiness (5t,12t)  ^2^Explainability (9d,12t)  Usefulness (9d,2t,3s)  Relevance (6s)  Reliability (12t)  Accuracy (4t)  Importance  Legibility  Format  Conciseness  Completeness  Timeliness  Data entry methods | Empathy (12t,3s,7s)  Quick responsiveness  Assurance Follow up service  Technical support |

(Number, Letter) in the table after the elements for example (1s): The number is a reference to the articles which the element was found (Articles referencing in Table 3 in the manuscript). The letters (d,t,s) are abbreviations for the type of AI systems investigated in the article (d: Diagnostics and preventive AI system, t: Treatment selection AI system, s: Self-help AI system). For example (1c) = Article 1 (as in table 3), the AI system type is Self-help AI.
^1,2^ : elements added from the initial inductive analysis.

**Reference**

1. Yusof MM, Kuljis J, Papazafeiropoulou A, Stergioulas LK. An evaluation framework for Health Information Systems: human, organization and technology-fit factors (HOT-fit). International journal of medical informatics. 2008 Jun;77(6):386-98. PMID: 17964851. doi: 10.1016/j.ijmedinf.2007.08.011.
